# Applications to Produce Local Anesthesia

**Published:** 1897-12

**Authors:** 


### Applications to Produce Local Anesthesia.

Le Gerant and E. Pierre (*Semaine Medecale*) employ the following:

| | |
|---|---|
| Chloroform - - - - - - | 10 parts |
| Ether - - - - - - | 15 parts |
| Menthol - - - - - - | 1 part |

The anesthesia resulting from this application lasts about five minutes.

